# Automated localization of mandibular landmarks in the construction of mandibular median sagittal plane

**DOI:** 10.1186/s40001-024-01681-2

**Published:** 2024-01-29

**Authors:** Yali Wang, Weizi Wu, Mukeshimana Christelle, Mengyuan Sun, Zehui Wen, Yifan Lin, Hengguo Zhang, Jianguang Xu

**Affiliations:** 1 Key Lab. of Oral Diseases Research of Anhui Province, College & Hospital of Stomatology, Anhui Medical University, 81 Meishan Road, Hefei, 230032 China; 2 Department of Orthodontics, Affiliated Hospital of Stomatology, Anhui Medical University Hefei, 69 Meishan Road, Hefei, Anhui China; 3 Paediatric Dentistry and Orthodontics, Faculty of Dentistry, The University of Hong Kong, Hong Kong SAR, China

**Keywords:** Deep learning, Mandibular median sagittal plane, Mandible segmentation, 3D imaging

## Abstract

**Objective:**

To use deep learning to segment the mandible and identify three-dimensional (3D) anatomical landmarks from cone-beam computed tomography (CBCT) images, the planes constructed from the mandibular midline landmarks were compared and analyzed to find the best mandibular midsagittal plane (MMSP).

**Methods:**

A total of 400 participants were randomly divided into a training group (*n* = 360) and a validation group (*n* = 40). Normal individuals were used as the test group (*n* = 50). The PointRend deep learning mechanism segmented the mandible from CBCT images and accurately identified 27 anatomic landmarks via PoseNet. 3D coordinates of 5 central landmarks and 2 pairs of side landmarks were obtained for the test group. Every 35 combinations of 3 midline landmarks were screened using the template mapping technique. The asymmetry index (AI) was calculated for each of the 35 mirror planes. The template mapping technique plane was used as the reference plane; the top four planes with the smallest AIs were compared through distance, volume difference, and similarity index to find the plane with the fewest errors.

**Results:**

The mandible was segmented automatically in 10 ± 1.5 s with a 0.98 Dice similarity coefficient. The mean landmark localization error for the 27 landmarks was 1.04 ± 0.28 mm. MMSP should use the plane made by B (supramentale), Gn (gnathion), and F (mandibular foramen). The average AI grade was 1.6 (min–max: 0.59–3.61). There was no significant difference in distance or volume (*P* > 0.05); however, the similarity index was significantly different (*P* < 0.01).

**Conclusion:**

Deep learning can automatically segment the mandible, identify anatomic landmarks, and address medicinal demands in people without mandibular deformities. The most accurate MMSP was the B-Gn-F plane.

**Supplementary Information:**

The online version contains supplementary material available at 10.1186/s40001-024-01681-2.

## Introduction

Mandibular deviation is a common deformity in orthodontic clinics and may be caused by differences in the size and shape of the mandible or by positional deviation of the mandible [1, 2]. According to the different mechanisms of deviation, the solution also varies. Therefore, accurate identification of the deviation mechanism is crucial for treatment. The craniomaxillary median sagittal plane (CMSP) was used as the sagittal plane for assessing mandibular symmetry in traditional methods; however, this plane has limitations when the position of the mandible changes, such as during rotation or translation [1, 3].

With the rapid development of computer and 3D reconstruction technology, 3D measurement methods have more advantages than two-dimensional (2D) methods for asymmetry evaluation [2, 4]. The methods for craniofacial symmetry analysis are based mainly on anatomical landmarks, original-mirror alignment, and deep learning algorithms. Conventional anatomic landmark approaches used to be the main option for evaluating craniofacial asymmetry. A reference plane is frequently created by dividing the lines between bilateral landmarks or by connecting median landmarks, which determine both sides of 2D or 3D quantitative measurements. Several studies have analyzed the planes constructed by B, G, and Me to explore the differences between the two sides of the mandible [5, 6]. The accuracy of this plane was not verified. A mandibular-specific sagittal midline plane for 3D mandibular analysis. This study was limited by the use of dental landmarks, which are unreliable at atrophic alveolar ridges [7]. The original-mirror alignment was processed by overlapping and aligning the original and mirror models through algorithmic 3D spatial coordinate transformations, and the best sagittal plane was mathematically computed. The iterative closest point (ICP) approach was based on the least squares principle and iteratively matched the closest point with a minimum distance between the original and the matching mirror images [8, 9]. However, this method has several limitations. Point clouds for asymmetric regions were added to the computation, reducing the algorithm's accuracy [4, 8]. Like in the ICP algorithm, the Procrustes analysis (PA) algorithm selects the point cloud of the symmetric region and transforms the original model with the corresponding coordinates of the mirror model to achieve the best match [8, 9]. Nevertheless, it was still unavoidable for both approaches to add a subjective element of human intervention. Zhu et al. constructed a 3D facial median sagittal plane by implementing this approach with the weighted Procrustes analysis (WPA) algorithm. The average angle error between the WPA and the manually defined planes was 0.73^°^ ± 0.50^°^ [10]. The PRS-Net model based on the ShapeNet dataset automated the creation of the 3D point cloud data symmetry plane, which was used as an important reference for the automated generation of the face median sagittal plane [11].

**Fig.1:**
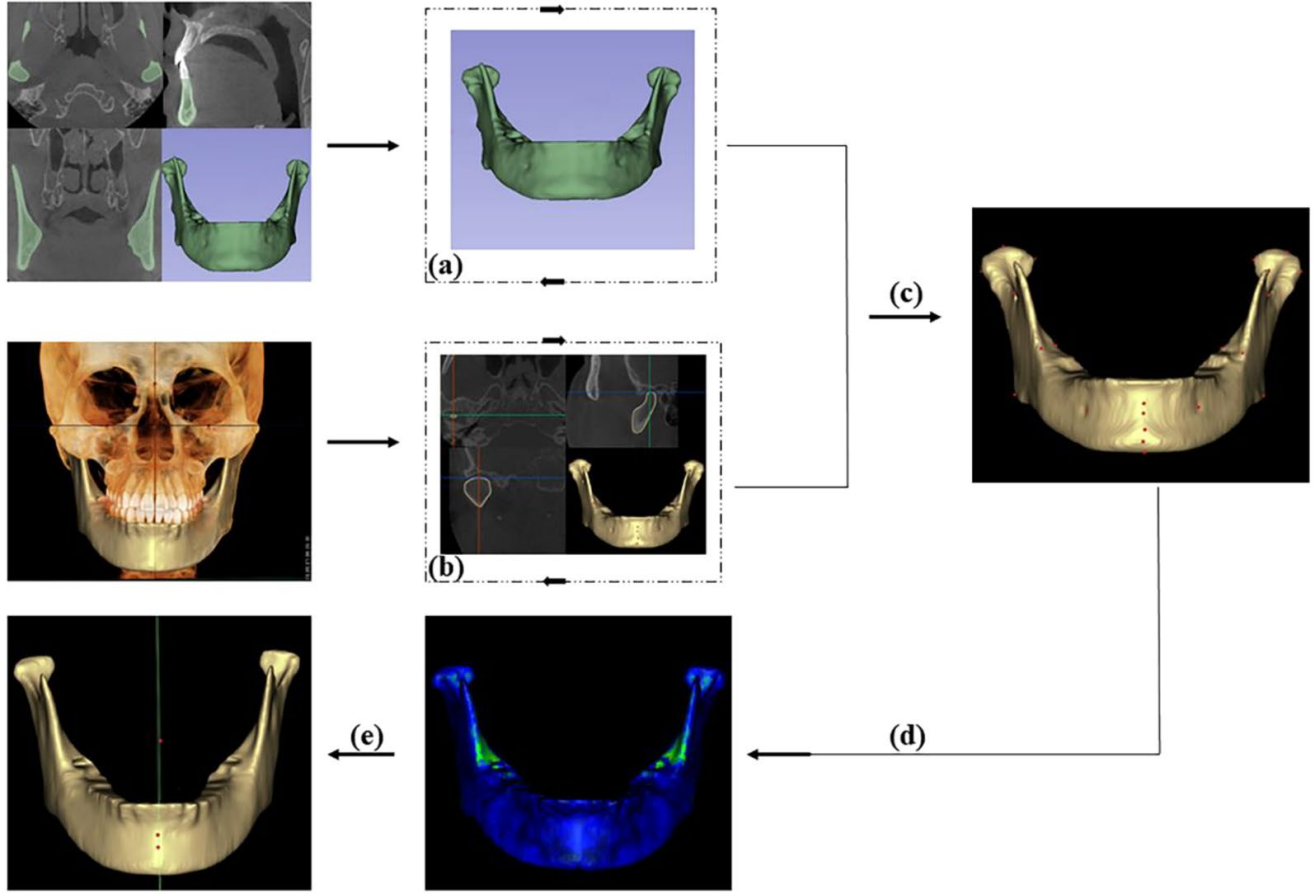
Automatic construction of MMSP by anatomical landmarks. **a** PointRend deep learning training; **b** PoseNet deep learning training; **c** mandible model; **d** template mapping technique; **e** MMSP was build

However, the use of deep learning techniques to construct sagittal planes is still in its infancy. In addition, research on constructing MMSP based on deep learning algorithms is rare. The purpose of this study was to automatically determine the mandibular midsagittal plane by segmenting it from CBCT images and accurately identifying its 3D landmarks using a deep learning algorithm (Fig. 1), thus providing a simple and accurate method for the clinical judgment of mandibular symmetry.

## Methods

### Data collection

Four hundred subjects aged 18 to 45 years were enrolled from the Anhui Medical University Stomatological Hospital from 2018 to 2022. All of the subjects were randomly divided into a training group (*n* = 360) and a validation group (*n* = 40). Exclusion criteria: mandible fractures or resorption, malformations, or incomplete CBCT images. Fifty craniofacial 3D images of morphologically normal people were obtained in the test group. Ethical approval was obtained from the Anhui Medical University Stomatological Hospital (PJ: T2020010). Written informed consent was obtained from all participants. The inclusion criteria were as follows: (1) aged 18 to 45 years (28 females and 22 males, mean age 28.02 ± 8.03 years); (2) 0^°^ < ANB angle < 4^°^, normal overjet, and overbite; (3) had no missing teeth except for the third molars; (4) had a complete CBCT scan covering the lower 2/3 of the maxillofacial region. Exclusion criteria: (1) systemic or genetic disorders that would influence mandible growth; (2) no cleft lip or palate, craniofacial syndromes, or deformities resulting from trauma or tumor; (3) no previous craniofacial surgery, facial fractures, or facial surgery.

### Image acquisition

All the CBCT images were acquired from a Meyer software (mDX-13STSP1A, Hefei, Anhui) at the following settings: 5 mA, 120 kV, exposure time of 20 s, scanning area of 23 × 18 cm, and focal point nominal of 0.5 × 0.5 mm. CBCT data were exported to Digital Imaging and Communications in Medicine (DICOM) files and imported into MyDentViewer (a tool for dental image processing software that supports image browsing, measurement annotation, and digital implant simulation; version 1.0; Meyer, Hefei, Anhui) to reconstruct 3D images.

### PointRend and PoseNet development

**Fig.2:**
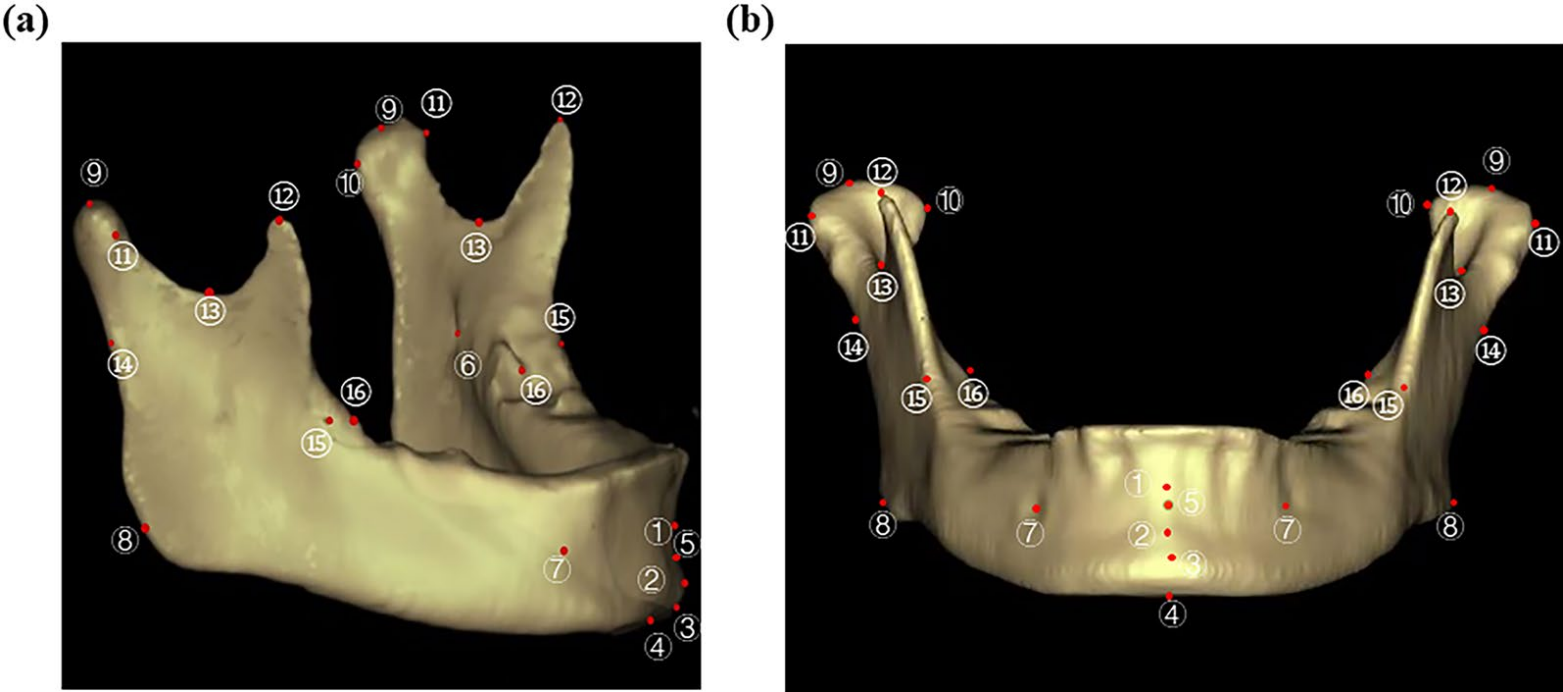
Anatomical landmarks of the mandible. **A**. right view; **B**. front view ① “supramentale” ② “pogonion” ③ “gnathion” ④ “menton” ⑤ “genial tubercle” ⑥ “fossa of mandibular foramen” ⑦ “mental foramen” ⑧ “gonion” ⑨ “condylion superius” ⑩ “condylion medialis” ⑪ “condylion lateralis” ⑫ “coronoid superius” ⑬ “sigmoid notch” ⑭ “ramus point” ⑮ “Jlat” ⑯ “Jmed”

The PointRend algorithm architecture for automatic mandible segmentation from CBCT images is shown in Additional file 1 (Figure 1). To avoid affecting mandibular measurements, the algorithm removes the crown and alveolar bone (detailed description in Additional file 1). The identification of the 3D landmarks of the mandible using the PoseNet algorithm is shown in Additional file 2. 3D mandibular and landmarks images are shown in Fig. 2.

### Template mapping

The template mapping technique ensured that 17,415 uniformly sampled quasi-landmarks were automatically identified on the entire mandibular surface [12]. Using the robust Procrustes alignment algorithm, the pointwise surface-to-surface distance between the original and mirror models in 3D space was calculated, which was expressed as the overall asymmetry index (AI). The position and severity of mandibular asymmetry are shown by a color map in millimeters [4, 13].

### Assessment of MMSP

**Fig. 3:**
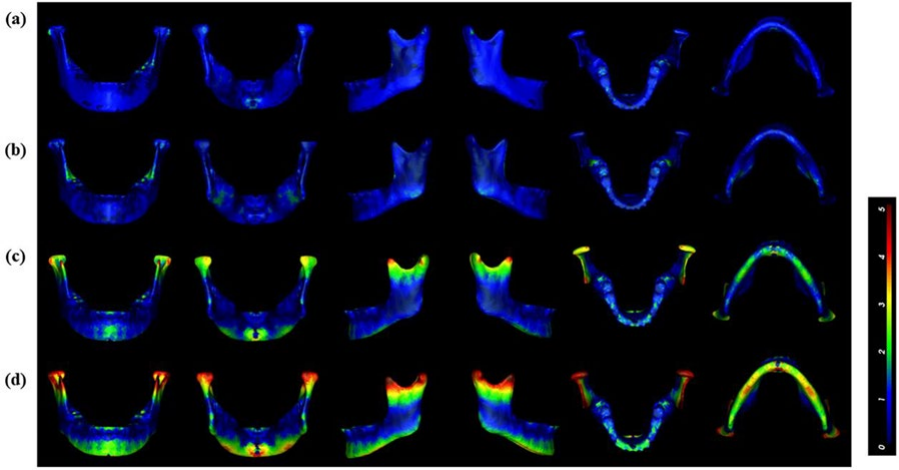
Mandibular asymmetry with the sagittal plane as the mirror plane; red indicates asymmetry greater than 4 mm, and dark blue indicates no asymmetry. **a** B-Gn-F: AI = 0.8; **b** B-Me-F: AI = 0.95; **c** B-Pog-F: AI = 1.34; **d** B-G-F:AI = 2.19

**Fig. 4:**
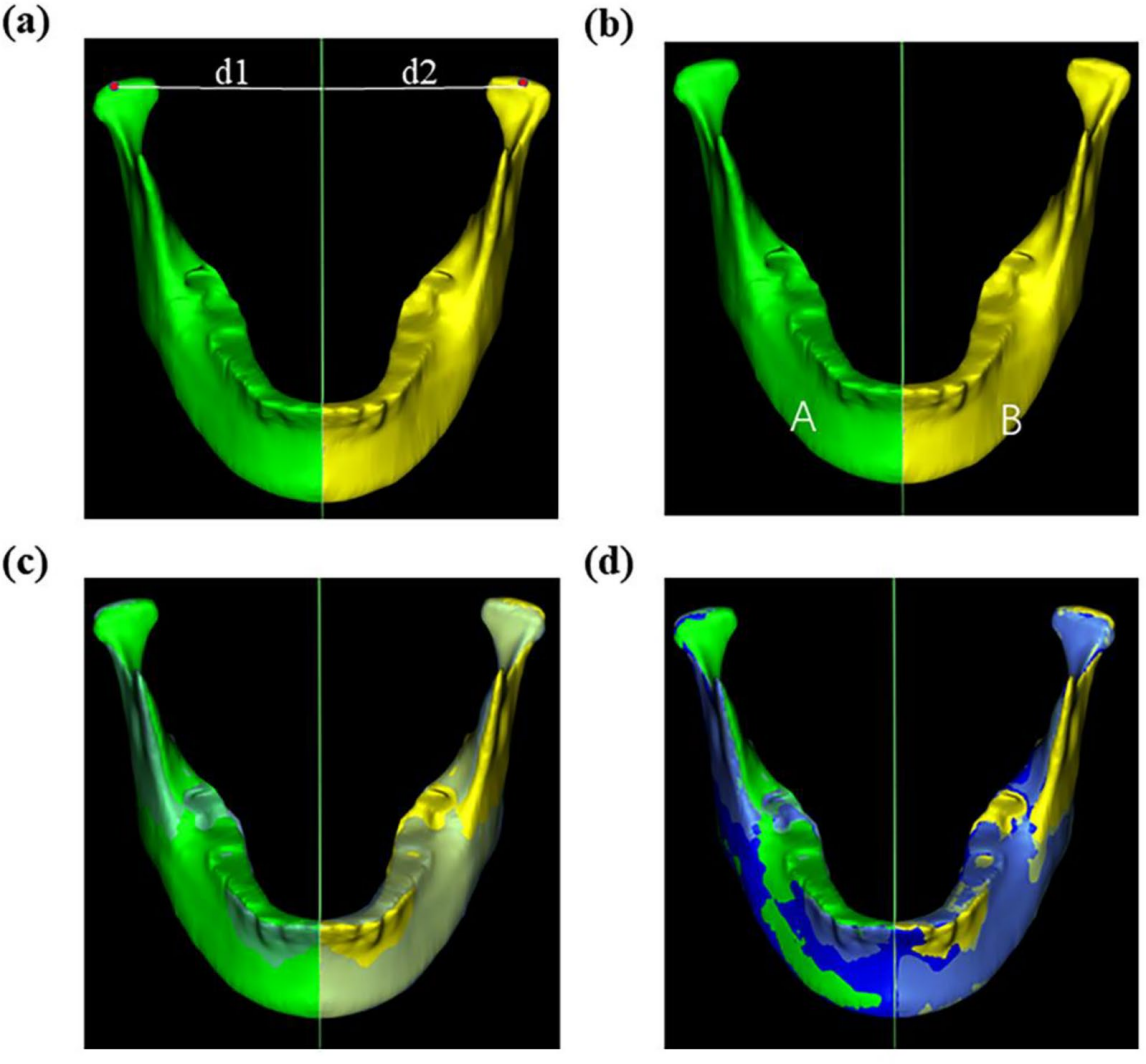
Green: left mandible A, yellow: right mandible B; **a** distance to MMSP (d1-d2); **b** difference in mandibular volume of two sides (**A**, **B**); **c** MMSP is a mirrored plane, superimposed on the left and right sides; **d** blue is the nonoverlapping area

Five central landmarks and the midpoint of 2 bilateral landmarks (mandibular foramen and mental foramen) composed 35 planes. Each plane was a mirror plane, and the first four planes with the lowest AIs were chosen (Fig. 3). The ideal MMSP was determined by clinical indices: distance, volume difference, and similarity index, in each of the four sagittal planes. Similarity index: sagittal plane mirroring of the left and right mandibles. Volumes were computed from nonoverlapping sections from overlapping images. The similarity index measures mandibular symmetry by comparing the overlapping volume to the total volume [2]. The similarity index was calculated as follows: 2 ∗ intersection (A, B)/ (A + B) (Fig. 4a, b, c, d).

### Statistical analysis

All the statistical analyses were performed with SPSS software (version 27.0; IBM, Armonk, NY), with *P* < 0.05 indicating statistical significance. A paired-sample t test was used to compare the mean differences between the reference plane and sagittal plane measurements.

## Results

### Model evaluation

Automatic mandibular segmentation took 10 ± 1.5 s, whereas the two operators averaged 2067.9 ± 425.91 s and 1987.9 ± 391 s, respectively (*P* = 0.183). The automated method exceeded the evaluations performed by two radiologists, achieving a Dice similarity coefficient of 0.98.

### Accuracy of landmarks identification

The PointRend method was used to locate anatomic landmarks within 0.5 s. The mean error of the 27 landmarks was 1.04 ± 0.28 mm; the error for the Me was the lowest (0.61 ± 0.18 mm), and that for the Conlat-R was the greatest (1.52 ± 0.28 mm) (Table 1).

**Table 1:** Landmark localization errors for the anatomical landmarks (mm)

Landmark	Mean error (mm)	Standard error of the mean (mm)
Me	0.61	0.18
B	0.43	0.28
Cor (right)	0.61	0.28
G	0.65	0.19
Pog	0.66	0.42
Conmed (left)	0.72	0.30
MF (left)	0.77	0.26
Sig (left)	0.79	0.22
Gn	0.79	0.39
MF (left)	0.80	0.22
Sig (right)	0.90	0.28
Jlat (right)	0.94	0.15
Cor (left)	1.17	0.35
Consup (right)	1.18	0.28
Go (left)	1.19	0.41
Jmed (left)	1.21	0.35
Jmed (right)	1.22	0.19
Conmed (right)	1.22	0.38
Go (right)	1.23	0.13
RP (right)	1.29	0.38
Consup (left)	1.30	0.24
Conlat (left)	1.32	0.34
F (right)	1.37	0.13
F (left)	1.39	0.21
RP (left)	1.41	0.44
Jlat (left)	1.45	0.27
Conlat (right)	1.52	0.28

### Plane evaluation

The first four planes of AI were B-Gn-F (1.6), B-Me-F (1.95), B-Pog-F (1.97), and B-G-F (2.15) (Table 2). The B-Gn-F plane has the smallest error among the four planes. Except for Cor (*P* < 0.05), Go (*P* < 0.05), and RP (*P* < 0.01), the other seven landmarks showed no statistically significant differences in distance (*P* > 0.05) (Table 3). The difference in volume between the two sides was not statistically significant (*P* = 0.671). Nevertheless, the symmetry index was significantly different (*P* < 0.01). The other 3 planes were significantly different from the reference plane except for the difference in mandibular volume on both sides (Table 4).

**Table 2:** Asymmetry index (AI) of planes

Landmark1	Landmark2	Landmark3	Median (IQR)	Min–Max
B	Gn	F	1.6(1.53–1.92)	0.59–3.61
B	Me	F	1.95(1.81–2.34)	1.05–3.61
B	Pog	F	1.97(1.75–2.19)	0.73–5.05
B	G	F	2.15(1.88–2.81)	0.68–5.38
B	F	MF	2.34(2.22–3.11)	0.69–4.34
..	..	..	..	..
..	..	..	..	..
B	G	Me	7.49(7.24–10.3)	1.56–19.97
..	..	..	..	..
..	..	..	..	..
B	G	MF	12.57(9.45–16.76)	1.58–41.71
B	Gn	Me	12.97(11.67–15.82)	1.34–37.4
G	Me	MF	17.78(18.44–25.32)	1.81–36.82
B	Pog	Gn	18.76(17.25–25.92)	0.94–53.46
Pog	Gn	Me	21.15(18.49–33.68)	1.7–64.04

*IQR* interquartile range (25th, 75th percentile); *Min* minimum; *Max* maximum; *AI* asymmetry index

**Table 3:** Mean distance difference from landmarks to MMSP

Landmark	Reference plane(mm)	B-Gn-F(mm)	B-Pog-F(mm)	B-Me-F(mm)	B-G-F(mm)
Consup	1.46 ± 0.97	1.56 ± 1.21 *P* = 0.628	2.9 ± 1.85**	2.93 ± 2.41**	3.07 ± 2.91**
Conmed	1.3 ± 1.07	1.36 ± 1.1 *P* = 0.739	2.38 ± 1.71**	2.25 ± 2.21**	2.88 ± 2.8**
Conlat	1.38 ± 1.12	1.72 ± 1.35 *P* = 0.085	2.58 ± 1.72**	2.69 ± 2.27**	2.74 ± 2.49**
Sig	0.84 ± 0.59	1.05 ± 0.88 *P* = 0.121	1.8 ± 1.23**	1.8 ± 1.75**	2.2 ± 2.04**
Cor	0.93 ± 0.68	1.39 ± 1.04 *	2.88 ± 1.95**	2.71 ± 2.5**	3.66 ± 3.19**
Go	1.43 ± 1.07	1.85 ± 1.38 *	1.99 ± 1.78*	1.98 ± 1.7*	2.13 ± 1.74*
RP	0.74 ± 0.62	1.22 ± 0.88 **	1.38 ± 0.89**	1.42 ± 1.19**	1.5 ± 1.17**
MF	1.97 ± 1.51	0.99 ± 0.73 *P* = 0.537	1.06 ± 0.74 *P* = 0.260	1.1 ± 0.69 *P* = 0.197	1.05 ± 0.67*
Jlat	0.98 ± 0.66	0.91 ± 0.65 *P* = 0.131	1.14 ± 0.81 *P* = 0.364	1.17 ± 0.98*	1.38 ± 1.13*
Jmed	1.26 ± 1.12	1.46 ± 1.23 *P* = 0.773	1.42 ± 1.24**	1.54 ± 1.35**	1.67 ± 1.39**
F	–	0 ± 0	0 ± 0	0 ± 0	0 ± 0
Mean	1.23 ± 0.37	1.23 ± 0.49 *P* = 0.893	1.78 ± 0.75 *P* = 0.066	1.78 ± 1.16 *P* = 0.057	2.03 ± 1.4*

^*^P < 0.05

^**^P < 0.01

**Table 4:** Absolute values of volume and similarity index difference for MMSP

Plane	Volume (mm^3^)	*P value*	Similarity index	*P value*
Reference	1066.39 ± 781.95	–	0.86 ± 0.04	–
B-Gn-F	1106.06 ± 781.73	0.671	0.81 ± 0.07	**
B-Pog-F	1124.67 ± 774.83	0.560	0.76 ± 0.1	**
B-Me-F	1148.88 ± 840.56	0.438	0.78 ± 0.1	**
B-G-F	1131.17 ± 794.73	0.537	0.74 ± 0.12	**

^*^P < 0.05

^**^P < 0.01

## Discussion

In recent years, artificial intelligence has been increasingly used in the field of medicine. In the field of orthodontics, the development of automatic cephalometric analysis is urgently needed [14]. We combined deep learning for mandibular segmentation, automated localization of landmarks, and automated construction of MMSP to enhance clinical efficiency and reveal the area and extent of mandibular asymmetry more precisely and intuitively. We demonstrated that the B-Gn-F plane is closest to the sagittal plane of the mandible.

Accurate segmentation of the mandible on CBCT is necessary for 3D analysis of the mandible. Semiautomatic segmentation methods based on threshold and region-growing algorithms are time-consuming and subjective and cannot be widely used in clinical practice [15–17]. Deep learning algorithms are more effective and accurate than traditional segmentation methods. Recently, medical image segmentation has employed artificial intelligence image segmentation techniques, and a convolutional neural network (CNN), a deep learning algorithm, has been commonly employed to analyze images [18]. CNNs learn task-specific features from data and are effective at image categorization, target identification, and recognition [17, 19]. Verhelst et al. constructed a 3D model of the mandible using a layered 3D U-Net architecture deep learning algorithm for direct segmentation of high-resolution CBCT images in 17 s, a 71.3-fold reduction compared to semiautomated segmentation [17]. Robert et al. created in-house segmentation software that increased the segmentation accuracy to 94.2% while decreasing the segmentation time to 2 min and 3 s. Segmenting skull bones with Mimics software provided Dice similarity coefficients of 0.924 and 0.949 for the maxilla and mandible, respectively, compared to the ground truth [20]. However, in this study, the time needed for mandibular segmentation by the PointRend algorithm was reduced to 10 ± 1.5 s, and the Dice similarity coefficient was 0.98 [17]. PointRend (point rendering) based on CNNs is an iterative segmentation technique proposed for efficient picture target recognition and segmentation [21, 22]. Inspired by computer graphics image rendering, this novel image segmentation method solves pixel labeling tasks and over- and under-sampling issues by treating image segmentation as a rendering problem and generating high-resolution segmentation masks [21–23]. With improved segmentation efficiency and application in CBCT 3D data, forward processing was performed directly on the whole sample, and the overall features of the mandible were better recognized from 3D space. The mandible in the image slices exhibited similar localized features to those of some regions of the maxilla and teeth. Notably, a clearer and more complete mandibular boundary can be extracted by iterative upsampling of only the edge points at the end of the model using the multilevel information of the network without affecting most of the foreground pixels.

3D anatomical landmarks reflect the morphological characteristics of the mandible and are the basis of 3D mandibular analysis. Manual landmark localization relies on doctors' clinical expertise and is tedious [24, 25]. The You-Only Look-Once version 3 (YOLOv3) algorithm was applied to 1028 cephalograms to automatically identify 80 cephalometric landmarks with a manual landmark average error of 1.46 ± 2.97 mm [25]. Zhang et al. simultaneously achieved the joint bone segmentation and landmark digitization (JSD) framework by context-guided fully convolutional networks (FCNs) with an average error of 1.1 mm for 15 anatomical landmarks [26]. In this study, we used the PoseNet algorithm [29], which has high expansibility and can accurately locate key points in 3D CBCT images without adding additional structure or computations to the model. On average, the error of the 27 landmarks was 1.04 ± 0.28 mm, while the clinical acceptability was 2 mm [24, 25, 27]. The mean error of the central landmarks was 0.63 ± 0.29 mm, which was smaller than that of the lateral landmarks 1.13 ± 0.28 mm, probably because the central landmarks were more accurate and reliable [24]. According to Schlicher et al., landmarks with a distinct anatomical structural contour showed fewer errors than landmarks that were located on curves, which also supported this result [28].

Previous studies have shown that central markers are more accurate than bilateral markers when the sagittal plane of the face is used. However, for the mandible, the bony markers at the mandible are very close together [29]. The reliability and stability of the MF and F points were high, and both sides of the structure were symmetrical, with no significant difference [7, 30, 31]. The F point was located on the posterior region of the mandible, and the fact that 4 planes were screened in this experiment provided additional evidence that this point is involved in the composition of the sagittal plane with a high degree of stability. Therefore, in this study, the MF and F points were added to form the sagittal plane.

The template mapping approach based on the MeshMonk algorithm was used to automatically assess the AIs of 35 planes. Technical template mapping and 3D surface-to-surface deviation analysis have become important scientific tools in orthodontics for studying changes in skeletal morphology [9, 32, 33]. This method was used in this study to mirror and superimpose a mandibular model onto a 3D color map to precisely identify morphological asymmetries. This approach, which was more intuitive and accurate than linear and angular measurements, assesses the asymmetry of the whole mandibular surface [4]. The ICP was not selected as a reference plane because although both methods use the root-mean-square value, the ICP algorithm calculates the closest distance between the original model and the mirror model, whereas the template mapping method calculates the distance between the original model and the mirror corresponding quasi-landmarks, which has the advantages of more points, intelligence, and good correspondence relationships [9]. When the mandible was heavily asymmetrical, the precision of the ICP algorithm was reduced [4]. Many previous studies used the B-G-Me plane as the MMSP to study the symmetry of mandibles [5, 6]. The results of the present study showed that the AI of the B-G-Me plane was 7.49 (Table 2), which differed significantly from the results obtained for the B-Gn-F plane (AI = 1.6). When the 3 points were spread to form a larger and broader triangle, the stability of the plane increased [34]. In contrast, 3 points in the B-G-Me plane were located at the mandibular symphysis, close enough to make the plane unstable.

Mandibular asymmetry is a complicated condition that is categorized into morphological and spatial structural differences between two sides [35]. On both sides of the mandible, the difference in volume between the 4 planes and the reference plane was not statistically significant (*P* > 0.05; Table 4). Although volumetric data are often used to compare differences between two sides of the mandible, these data do not allow for quantitative assessment of symmetrical and asymmetrical regions [36]. The similarity index and nonoverlapping volume can reflect both morphological and structural differences in the mandible [2]. Significant differences were found between the B-Gn-F plane and the reference plane (*P* < 0.01) (Table 4). This was probably because the algorithmic analysis examined the shape of the mandible, while the anatomical landmarks method simply looked at the structure. However, the mean value of the overall similarity index was closest to that of the reference plane. Therefore, it is reasonable to use the B-Gn-F plane as the MMSP in the clinical analysis of the mandible.

To assess mandibular asymmetry, landmarks-based analyses were applied. In this method, distance, volumetric data, similarity index, surface-to-surface deviation analysis, and template mapping techniques were combined to screen for relatively accurate MMSP data, facilitating the identification of regions affected by asymmetry. The limitations of this study are that the automated localization accuracy of severe mandibular deformity data was not evaluated. Hence, only adults with mandible symmetry were studied, while other asymmetry types were not confirmed. The MyDentViewer software is the unopen source and is currently used for collaborative research.

## Conclusions

In this study, automated segmentation of the mandible and localization of anatomical landmarks were used to realize automatic cephalometric analysis. Furthermore, the B-Gn-F plane can be used for clinical application because of the symmetry of the mandibular structure.

The benefits of artificial intelligence go beyond shortening the time needed to obtain asymmetric information. Combining deviation analysis with artificial intelligence provides a more efficient workflow, and accurate quantification of mandibular asymmetry will help orthodontists and surgeons better understand asymmetry and guide treatment planning. Color-coded charts not only are an important tool for diagnosis, but also allow patients and parents to easily understand asymmetry.

### Supplementary Information

**Additional file 1: Figure S1.** Framework of Point-Rend deep learning segmentation of mandible.

**Additional file 2: Table S1.** Description of mandibular landmarks.

## Data Availability

The datasets used and analyzed during the current study are available from the corresponding author upon reasonable request.

## Abbreviations

CBCT: Cone beam computed tomography
MMSP: Mandibular midsagittal plane
AI: Asymmetry index
3D: Three-dimensional
2D: Two-dimensional
CMSP: Craniomaxillary median sagittal plane
ICC: Intraclass correlation coefficient
ICP: Iterative closest point
WPA: Weighted Procrustes analysis
