# Supplementary material for: Automated localization of mandibular landmarks in the construction of mandibular median sagittal plane
Source: Eur J Med Res. 2024 Jan 29;29:84. doi: 10.1186/s40001-024-01681-2 (PMC10823719; doi:10.1186/s40001-024-01681-2)
Supplement: Supplementary file 1 — Additional file 1: Figure S1. Framework of Point-Rend deep learning segmentation of mandible. [file 40001_2024_1681_MOESM1_ESM.docx]

Step 1: Manual Segmentation

Two expert radiologists manually segmented the mandibular images from the 50 CBCT images with open-source software (3DSlicer; version 5.2.2; <http://www.slicer.org>). Manual segmentation was considered the gold standard of 3D model segmentation for some regions with low image density or similar to other structures without definite boundaries [1, 2].

Step 2: PointRend framework

The iterative upsampling layer was mainly manifested by looping through the following steps when the output mask resolution was less than the input segmentation rate. The framework of automatic segmentation is shown in Figure 1.

1) The coarse prediction results coarse_ prediction_ I for the current iteration can be obtained by upsampling the current output mask by a factor of 2 (usually with bilinear interpolation);

2) The mask resulted in the selection of N “difficult pixel points”, i.e., points in the mask where the predicted label was likely to be different from the surrounding pixel points (in this case, basically edge points).

3) For each difficult pixel point, its representation vector was computed for the next prediction of the point's label, which consisted of two parts: one was the low-level features (fine-grained features), obtained by bilinear interpolation on the low-level feature map using the point's coordinates, and the other was the high-level features (the coarse prediction results obtained in step 1).

4) A small MLP layer was used to compute a new prediction for the "representation vector” and update coarse_ prediction_ I to obtain the coarse prediction _ prediction_ I for the next iteration. Each conv1*1 served only one "hard pixel".

Step 3: Model Evaluation

The results of automatic segmentation of the 50 images were compared with those of manual segmentation by two radiologists, and the time of completion of segmentation was recorded for each data set. The Dice similarity coefficient (DSC) is widely used to evaluate the performance of image segmentation algorithms [3, 4].

**References**

1. Ilesan RR, Beyer M, Kunz C, Thieringer FM: **Comparison of Artificial Intelligence-Based Applications for Mandible Segmentation: From Established Platforms to In-House-Developed Software**. *Bioengineering (Basel)* 2023, **10**(5).

2. Lo Giudice A, Ronsivalle V, Grippaudo C, Lucchese A, Muraglie S, Lagravere MO, Isola G: **One Step before 3D Printing-Evaluation of Imaging Software Accuracy for 3-Dimensional Analysis of the Mandible: A Comparative Study Using a Surface-to-Surface Matching Technique**. *Materials (Basel)* 2020, **13**(12).

3. Qiu B, van der Wel H, Kraeima J, Glas HH, Guo J, Borra RJH, Witjes MJH, van Ooijen PMA: **Automatic Segmentation of Mandible from Conventional Methods to Deep Learning-A Review**. *J Pers Med* 2021, **11**(7).

4. Qiu B, van der Wel H, Kraeima J, Hendrik Glas H, Guo J, Borra RJH, Witjes MJH, van Ooijen PMA: **Robust and Accurate Mandible Segmentation on Dental CBCT Scans Affected by Metal Artifacts Using a Prior Shape Model**. *J Pers Med* 2021, **11**(5).


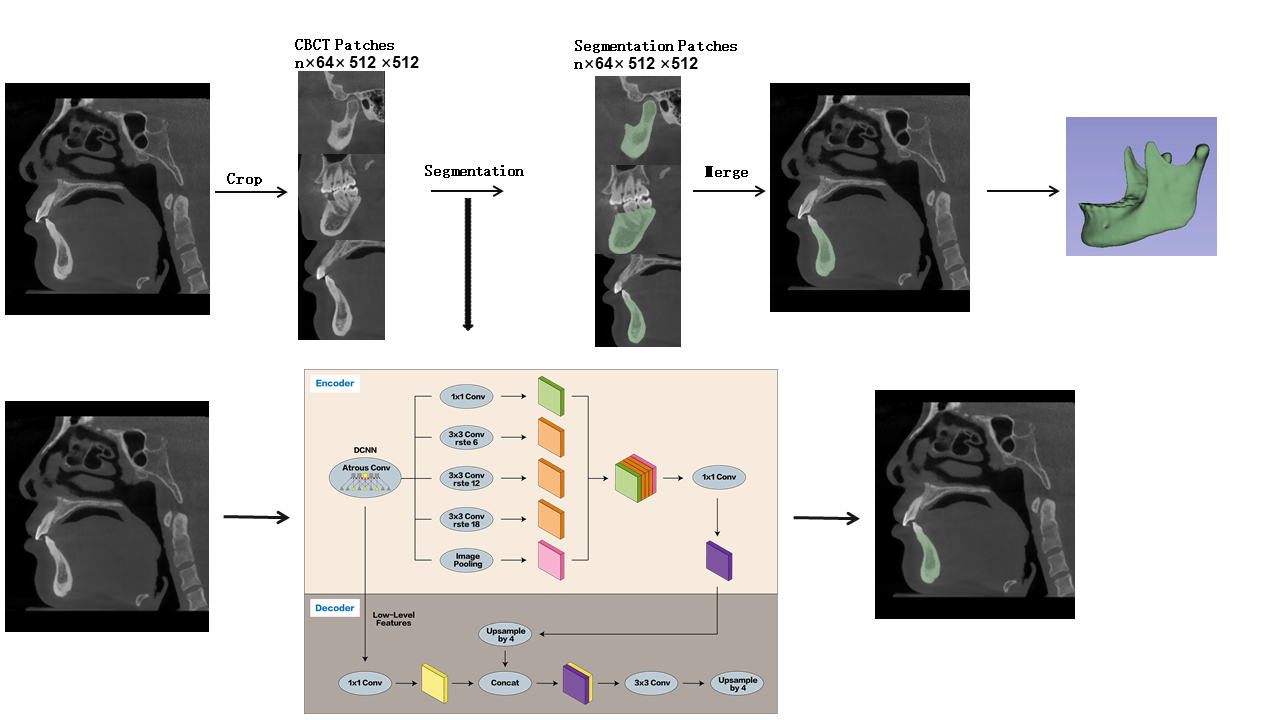


Figure 1. Framework of Point-Rend deep learning segmentation of mandible.
