# Supplementary material for: Automated localization of mandibular landmarks in the construction of mandibular median sagittal plane
Source: Eur J Med Res. 2024 Jan 29;29:84. doi: 10.1186/s40001-024-01681-2 (PMC10823719; doi:10.1186/s40001-024-01681-2)
Supplement: Supplementary file 2 — Additional file 2: Table S1. Description of mandibular landmarks. [file 40001_2024_1681_MOESM2_ESM.docx]

Step 1: Manual Landmarking

A total of 27 landmarks, including 5 central landmarks and 22 bilateral landmarks, were manually landmarked by 2 operators: operator 1, who was a trained orthodontist with 3 years of clinical experience, and operator 2, who was a radiologist with 10 years of experience in radiology. Each operator performed manual landmarking three times at an interval of 3 days. The intragroup correlation coefficients (ICC) for X, Y, and Z of each marker point were greater than 0.99. The ground truth is compared with the coordinates generated by the PoseNet algorithm. A detailed description of the landmark definitions is given in Table 1.

Step 2: PoseNet model training

The architecture used a PoseNet algorithm extended from the two-dimensional PoseNet, and the dimension was extended to PoseNet-3d, which was suitable for three-dimensional medical images. Since the studied fixed points cover the entire spatial range of the data, the receptive field of the model had a crucial impact on the fixed-point accuracy. Generally, to increase the receptive field, researchers would add multiple pooling layers to the network; however, this would inevitably lead to the loss of detailed information, so the study discarded some pooling operations in the upsampling process and replaced them with the null convolution to reduce the size of the feature map and increase the receptive field at the same time. Finally, without increasing the extra structure and computation of the model, the needed key points in the 3D CBCT image can be accurately localized, taking both efficiency and accuracy into account.

Step3: Metrics

The error between the manual and PoseNet algorithm was calculated according to the Euclidean distance equation [1].

**References**

1. Weingart JV, Schlager S, Metzger MC, Brandenburg LS, Hein A, Schmelzeisen R, Bamberg F, Kim S, Kellner E, Reisert M *et al*: Automated detection of cephalometric landmarks using deep neural patchworks. *Dentomaxillofacial Radiology* 2023.

Table 1. Description of mandibular landmarks

| Landmarks | Definition |
| --- | --- |
| Unilateral |  |
| B (supramentale) | The midpoint of the greatest concavity on the anterior border of the symphysis |
| Pog (pogonion) | The most anterior midpoint on the symphysis |
| Gn (gnathion) | The most caudal point along the curvature  of the symphysis of the mandible in the midsagittal plane |
| Me(menton) | The most inferior midpoint on the symphysis |
| G (genial tubercle) | The midpoint on genial tubercle |
| Bilateral |  |
| F (fossa of mandibular foramen) | The most inferior point of the fossa of the mandibular foramen |
| MF (mental foramen) | The entrance of the mental foramen |
| Go(gonion) | The most prominent point of the lateral mandibular angle |
| Consup (condylion superius) | The most superior point of the condylar head |
| Conmed (condylion medialis) | The most medial point of the condylar head |
| Conlat (condylion lateralis) | The most lateral point of the condylar head |
| Corsup (coronoid superius) | The most superior point of the coronoid process |
| Sig (sigmoid notch) | The lowest point of the semilunar incisura of sigmoid notch |
| RP (ramus point) | Posterior border of the left mandibular ramus |
| Jlat | The most lateral and deepest point of the curvature formed at the junction of the mandibular ramus and body |
| Jmed | The most medial and deepest point of the curvature formed at the junction of the mandibular ramus and body |
| Generated by computer software |  |
| Fmid (fossa of mandibular foramen midpoint) | Midpoint between the left and right F |
| MFmid (mental foramen midpoint) | Midpoint between the left and right MF |
